# Vaccines, Politics and Mandates: Can We See the Forest for the Trees? Comment on "Convergence on Coercion: Functional and Political Pressures as Drivers of Global Childhood Vaccine Mandates"

**DOI:** 10.34172/ijhpm.2022.7572

**Published:** 2022-10-22

**Authors:** Noni E. MacDonald, Ève Dubé, Jeannette Comeau

**Affiliations:** ^1^Department of Paediatrics, Faculty of Medicine, Dalhousie University, Halifax, NS, Canada.; ^2^Direction des Risques Biologiques et de la Santé au Travail, Institut National de Santé Publique du Québec, Québec, QC, Canada.

**Keywords:** Vaccine Mandates, Vaccine Hesitancy, Vaccine Acceptance, Vaccination Politics, COVID Vaccines, Childhood Vaccines

## Abstract

Under-vaccination is a complex problem that is not simple to address whether this is for routine childhood immunization or for coronavirus disease 2019 (COVID-19) vaccination. Vaccination mandates has been one policy instrument used to try to increase vaccine uptake. While the concept may appear straight forward there is no standard approach. The decision to shift to a more coercive mandated program may be influenced by both functional and/or political needs. With mandates there may be patient and/or public push back. Anti-mandate protests and increased public polarization has been seen with COVID-19 vaccine mandates. This may negatively impact on vaccine acceptance ie, be counterproductive, causing more harm than overall good in the longer term. We need a better understanding of the political and functional needs that drive policy change towards mandates as well as cases studies of the shorter- and longer-term outcomes of mandates in both routine and pandemic settings.

 Based upon fulsome scientific evidence, routine childhood vaccination has been recognized by modern public health as one of the most cost effective and long- lasting population health interventions that can decrease the burden of vaccine preventable diseases.^1^ Immunization is of such importance to health and wellbeing that it is well entrenched in the 2030 Sustainable Development Goals and contributes to 14 of the 17 goals.^2^ However, despite the clear and incontrovertible evidence of the laudable effect of vaccines on childhood morbidity and mortality, universal acceptance has remained elusive, even in high-income countries like the United States where there is growing evidence over the past two decades of vaccine refusal, vaccine mistrust and vaccine hesitancy (eg, ambivalence towards vaccine that can result in delayed vaccination or vaccine refusal) undermining uptake.^3^ Vaccine hesitancy is such a serious concern, that in 2019, the World Health Organization (WHO) listed vaccine hesitancy as one of the 10 major threats to global health: a problem in high-middle- and low-income countries (https://www.who.int/emergencies/ten-threats-to-global-health-in-2019). The coronavirus disease 2019 (COVID-19) pandemic has further emphasized the importance of vaccination. The emergency use listing for the first COVID-19 vaccines in late 2020 altered the course of the pandemic. By the end of 2021, COVID-19 vaccines had been estimated to have saved tens of millions of lives.^4^ However, the impact of vaccines on deaths overall was not the same across high-income countries (see Table). In these 15 selected high-income countries (Brazil: high-middle income) where COVID vaccine supply was not a major factor by mid-2021 once vaccine and roll out programs were in place, deaths per million varied markedly. The rates were higher in the countries where vaccine uptake lagged. Overall higher vaccine acceptance was associated with lower rates of deaths per million.

**Table:** COVID-19 Deaths Per Million and COVID-19 Percent Population Immunized in Selected Countries by Dates Noted

**Country**	**COVID-19 Deaths Per Million July 13, 2022**^a^	**COVID-19 Deaths Per Million ** **Pre Vaccine (March 2020 to December 2020)**^b^	**COVID-19 Vaccine Share People Complete Initial Protocol July 18, 2022**^c^	**COVID-19 Vaccine Share People Only Partially Vaccinated July 18, 2022**^c^
Australia	407	0.00	84%	86%
Brazil	3171	3.49	79%	86%
Canada	1111	3.81	82%	86%
Denmark	1111	1.95	82%	82%
France	2116	5.64	79%	81%
Germany	1708	6.80	76%	78%
Ireland	1509	0.83	81%	82%
Israel	1158	1.35	66%	72%
Italy	2844	10.87	81%	86%
Japan	250	0.33	82%	83%
Norway	635	0.45	75%	80%
Singapore	253	0.00	92%	92%
South Korea	477	0.22	86%	87%
The United Kingdom	2669	6.44	75%	80%
The United States	3100	8.02	67%	78%

Abbreviation: COVID-19, coronavirus disease 2019. ^a^July 13, 2022, https://www.statista.com/statistics/1104709/coronavirus-deaths-worldwide-per-million-inhabitants/. ^b^March 1, 2020, to December 18, 2020, selected from: https://ourworldindata.org/explorers/coronavirus-data-explorer. ^c^July 18, 2022, https://ourworldindata.org/covid-vaccinations.

 Under-vaccination is a complex problem that is not simple to address whether this is for routine childhood immunization or for COVID-19 vaccination.^5^ Mandates requiring seat belt and car seat use has contributed to a marked decrease in motor vehicle injury and deaths albeit more than legislation has been needed.^6^ Vaccination mandates has been one policy instrument used to try to increase vaccine uptake both for routine childhood immunization and for COVID-19 vaccines. While the concept may appear straight forward and simple at first glance, there are many twists and turns between the concept and its implementation that can affect its impact.^7^ There is no standard approach and wide variation in the ifs, whys, what’s, and when of mandatory vaccination implementation.^8^ Even the legal, regulatory, or ministerial decree frameworks used to support a mandatory program are not uniform. Hence, the how of mandatory childhood vaccination being put into practice has also much varied. The global exceptions for standardization are for vaccines required by international regulations (ie, yellow fever and polio) that are implemented and regulated in very similar ways.^7^

 Attwell and Hannah have examined in detail one puzzling aspect of mandatory childhood immunization implementation: the political underpinnings.^9^ In their report of four case studies, they delved into the nuances behind Italy, France, Australia, and California choosing to move to more coercive routine childhood immunization policies. These three countries and one state had quite different political orientations, motivation for considering mandating programs, differing contexts at the time of consideration as well as different immunization delivery system practices. The case studies well illustrate that in no two instances were the factors that lead to mandatory programs the same, including the depth of concerns about under-vaccination, all differed. However, worries about vaccine hesitancy alone was not the driver in moving to a more coercive program in any of the four cases studied. Attwell and Hannah emphasized the interaction of two major factors; the need to address technical immunization program related issues (ie, function pressures) and the threat to policy function (ie, political pressures) in the push towards shifting to a more coercive mandatory program. What all four cases illustrate well is the great appeal of mandatory; ie, mandatory programs can be targeted and implemented without huge costs or complex policy. This route may appear at a glance to be an easier strategy to increase vaccine uptake than many others advanced to address lower than desired vaccine acceptance rates.^9^ Of note, none of the four jurisdictions used the same compliance incentives or penalties, covered the same vaccines or age groups. One missing piece in this four-case political puzzle report, is what were the outcomes of these mandates initially, overtime, as contexts changed, and as other health measures competed for public health funding. Did a change in politics and /or context lead to changes in these policies?

 The COVID-19 pandemic is an example of change in context. These coercive policies for routine childhood immunization in these 3 countries and one state were all implemented in pre pandemic times. The COVID-19 pandemic has led early to other public health mandates influenced both by functional and political needs. Initial public health control measures centred not on vaccine mandates (as none were available) but on masks, handwashing, social distancing and stay at home requirements. Globally, some countries were agile in implementing these mandates while others were less so but for all, politics was a factor in the decisions.^10^ In a study of six high- and one high-middle income countries, political polarization widely undermined support for these public health policies aimed at controlling the spread of COVID-19.^11^ Polarization over pandemic measures reached the streets. Even before COVID-19 vaccines were available, protests especially against masks and stay at home measures began. Protesters disagreed with public health and the government on the significance of COVID-19. For them, COVID -19 was not a big enough threat to merit these perceived restrictive public health mandated infection control steps.^12^ The growth and sharing of COVID-19 conspiracy theories on social media helped fuel this distrust and enhance the belief that COVID-19 risk was either fictious or not important.^13^ The protests gathered further momentum when effective COVID-19 vaccines arrived, albeit initially targeted for use only in high-risk populations (healthcare workers and the older general populations), by bringing together the already well-organized anti-vaccination movement with the growing opposition to curtailment of individual freedoms movement with COVID-19 restrictions.^12^ The protests became even louder when vaccine mandates were implemented for international travel, for some work settings etc. We currently lack the nuanced case studies on mandates for COVID-19 nonpharmaceutical control measures and vaccine policies like the ones Attwell and Hannah have published for routine childhood immunization mandates.

 Concerns have been raised that COVID-19 vaccine requirements (eg, vaccine passport, mandatory vaccination of healthcare workers) may be counterproductive, causing more harm than good in the longer term.^14^ Bardosh and colleagues note that mandates may increase reactance to COVID-19 vaccination by further energizing anti-vaccine activism. The mask and vaccine mandates may lead to the undermining of trust in public health recommendations due to cognitive dissonance. For example, in many countries in 2022 changing public health recommendations for masks in the face of scientific evidence of their effectiveness and continued ongoing community COVID-19 spread has been confusing not only for the public but also for healthcare professionals who regularly wear masks to prevent transmission of respiratory viruses. Politics and political pressure appear likely to have played a role in these public health “unmasking” decisions in many settings, but the case studies are lacking. Further concerns raised by Bardosh and colleagues are the potential for stigmatization of groups singled out by vaccine mandates, the undermining of trust globally by adding support to conspiracy theories and the potential additive effects on polarization within communities and countries which may lead to an undermining of democracy. In many countries, those who support the collective good versus those focused on individual choice have moved even farther apart ie, more polarization as the COVID-19 pandemic has evolved.^11^ Public health ethics and integrity may also be undermined with the mandates and the response to these.^14^ For example, this would occur if the benefits to the public health interventions did not outweigh the restrictions to liberty and associated burdens ie, the principle of proportionality.^14^

 There are other specific immunization concerns that must also be considered beyond whether COVID-19 mandates will increase COVID-19 vaccine uptake. Will the emboldened COVID-19 anti-vaccine movement spill over to undermine community support for routine childhood vaccines? Will the impact of COVID-19 mandates on routine childhood vaccination differ between countries with and without pre-existing mandates for routine childhood immunization? Overall, will this lead to more parents and communities losing trust in routine childhood vaccines, in the immunization programs and in the governments that support them even if many still accept routine childhood immunization? Hence, will this eventually lead to underfunding of routine immunization programs and even more inequality in access if routine immunization is seen as less and less of being of consequence.

 The 2021 WHO/UNICEF Estimates of National Immunization Coverage (WUENIC) data has shown that the COVID-19 pandemic and its required control measures has already had a major impact on routine infant childhood immunization uptake rates globally in 2020 and 2021 (https://data.unicef.org/topic/child-health/immunization/, https://www.who.int/teams/immunization-vaccines-and-biologicals/immunization-analysis-and-insights/global-monitoring/immunization-coverage/who-unicef-estimates-of-national-immunization-coverage). More children are un or under-immunized than in 2019 ie, pre COVID. While the impact appears to have been greater in low- and low-middle income countries, high-income countries have not been spared. What is unknown, is whether these declines in routine childhood immunization can be readily rectified as the pandemic shifts to endemic and/or will the antivaccine movement’s growing noise and rhetoric further undermine acceptance. How will immunization programs respond; what will politicians do? Will more mandates for routine immunization be implemented or will some be rescinded or altered for mandates already in place? We also lack understanding of mandate politics and functions in low-income country settings for routine childhood immunization even though some have such programs in place.^10^

 Vaccine acceptance is a very complex area with many factors both internal to the person and external having influence. As Attwell and Hannah have shown in their three country one state case series, politics and functional program needs did influence the decision to shift routine childhood vaccine to a more coercive mandated program albeit with quite different program requirements. Unfortunately, many vaccine hesitancy models have not included immunization politics, laws, regulations, and program requirements as factors that can influence vaccine acceptance and hesitancy.^15^ In contrast, the Royal Society of Canada Vaccine Acceptance framework includes “polices, programs, practices and politics” in the healthcare and public health systems domain.^15^ The COVID-19 pandemic vaccine acceptance experience and implementation of mandates has very much highlighted the impact of politics. Vaccine mandates require more scrutiny and more research or globally we may be paying the price in more vaccine preventable disease outbreaks because of growing vaccine refusals.

## Ethical issues

 Not applicable.

## Competing interests

 Authors declare that they have no competing interests.

## Authors’ contributions

 NEM wrote the first draft and ED and JC reviewed and suggested revisions. All three reviewed and approved the final version.
